# Two communities, one highway and the fight for clean air: the role of political history in shaping community engagement and environmental health research translation

**DOI:** 10.1186/s12889-020-09751-w

**Published:** 2020-11-11

**Authors:** Linda Sprague Martinez, Noelle Dimitri, Sharon Ron, Neelakshi Hudda, Wig Zamore, Lydia Lowe, Ben Echevarria, John L. Durant, Doug Brugge, Ellin Reisner

**Affiliations:** 1 Macro Department, Boston University School of Social Work, Boston, MA 02215 USA; 2 Boston University School of Social Work, Boston, MA 02215 USA; 3 Metropolitan Area Planning Council, Boston, MA 02111 USA; 4 Department of Civil and Environmental Engineering, Tufts University, Medford, MA 02155 USA; 5 Somerville Transportation Equity Partnership, Somerville, MA 02145 USA; 6 The Chinatown Land Trust, Boston, MA 02111 USA; 7 The Welcome Project, Somerville, MA 02145 USA; 8 Department of Public Health Sciences, University of Connecticut School of Medicine, Farmington, CT 06030 USA

**Keywords:** Traffic related air pollution (TRAP); research and action, Community based participatory research, Public health action

## Abstract

**Background:**

This paper explores strategies to engage community stakeholders in efforts to address the effects of traffic-related air pollution (TRAP). Communities of color and low-income communities are disproportionately impacted by environmental threats including emissions generated by major roadways.

**Methods:**

Qualitative instrumental case study design was employed to examine how community-level factors in two Massachusetts communities, the City of Somerville and Boston’s Chinatown neighborhood, influence the translation of research into practice to address TRAP exposure. Guided by the Interactive Systems Framework (ISF), we drew on three data sources: key informant interviews, observations and document reviews. Thematic analysis was used.

**Results:**

Findings indicate political history plays a significant role in shaping community action. In Somerville, community organizers worked with city and state officials, and embraced community development strategies to engage residents. In contrast, Chinatown community activists focused on immediate resident concerns including housing and resident displacement resulting in more opposition to local municipal leadership.

**Conclusions:**

The ISF was helpful in informing the team’s thinking related to systems and structures needed to translate research to practice. However, although municipal stakeholders are increasingly sympathetic to and aware of the health impacts of TRAP, there was not a local legislative or regulatory precedent on how to move some of the proposed TRAP-related policies into practice. As such, we found that pairing the ISF with a community organizing framework may serve as a useful approach for examining the dynamic relationship between science, community engagement and environmental research translation. Social workers and public health professionals can advance TRAP exposure mitigation by exploring the political and social context of communities and working to bridge research and community action.

**Supplementary Information:**

**Supplementary information** accompanies this paper at 10.1186/s12889-020-09751-w.

## Background

In the United States (US) and abroad, communities of color and low-income communities are disproportionately impacted by environmental threats including noise and emissions generated by major roadways [1–3]. Pollution sources including manufacturing facilities, energy plants, highways, airports, and toxic waste sites are frequently situated in communities of color and low-income areas [1, 3–5]. Unfortunately, environmental policies are often not enforced at these polluted sites and resident complaints are inadequately addressed [1]. Resident action at the community level can be an effective mechanism for catalyzing change, particularly when efforts are informed by data [2]. As an approach, community-based participatory research (CBPR) combines inquiry and collective action and has gained popularity as a strategy for elevating health-related priorities in marginalized communities [6–8].

CBPR emerged on the health research scene nearly two decades ago and is seen as an effective approach to tackling inequities in health [9, 10]. CBPR is specifically designed to generate research that can inform policy and practice and catalyze change through the active engagement of community members in local decision-making processes [11–13]. In the context of environmental health research CBPR was catalyzed in part by funding support from the National Institute of Environmental Health and Science (NIEHS) [14]. Moreover, because it emerged in the context of health equity work, CBPR is specifically intended to drive changes to promote health equity and justice [13]. Moving from research to community action necessitates broad community engagement to shift public will. This is particularly true in the case of advancing environmental justice, which often involves policy change and modifications to the built environment, as well as shifts in both public and private sector practice. Communities are complex adaptive systems [15], and successful strategies to advance health, such as policy and practices to reduce inequities in pollution exposure, likely vary across and within communities [16]. This variability poses challenges for documenting and replicating best practices [16].

This paper explores strategies to engage community stakeholders in efforts to address a wide-spread environmental justice concern: the effects of traffic-related air pollution (TRAP). TRAP is defined as the complex mixture of gaseous and particulate pollutants present in tailpipe and non-tailpipe emissions from vehicles.

**Fig. 1:**
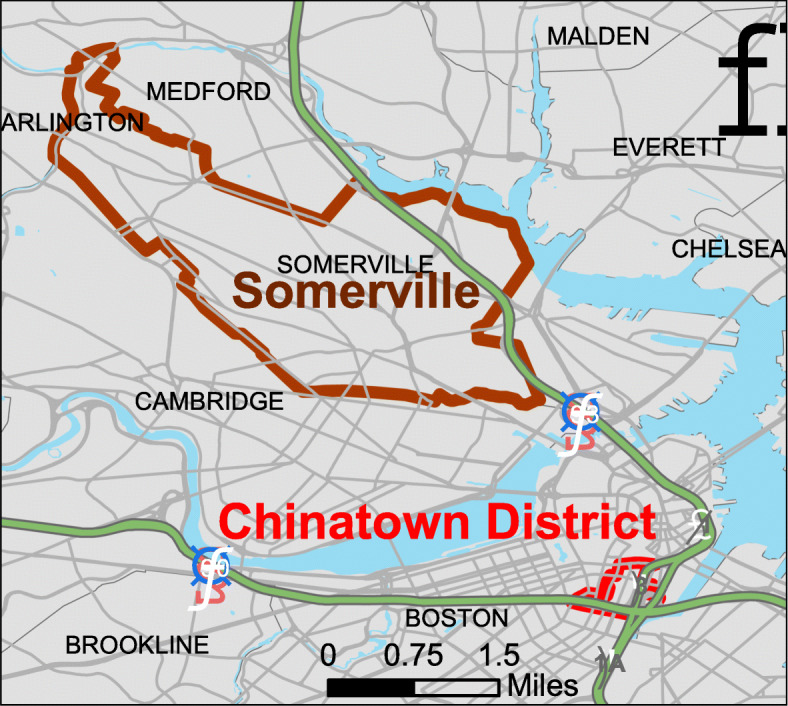
Map of Somerville and Chinatown. Layers for the map were obtained from mass.gov and map was generated using ArcMap10.5

The City of Somerville and the Boston, MA, Chinatown neighborhood (Somerville and Chinatown hereafter), just 3.5 miles apart, are two very different communities (see map Fig. 1).

Nonetheless, both share a history of marginalization and exposure to TRAP from Interstate-93 (I-93) and other highways. As noted in Table 1, both communities are disproportionately impacted by transportation and air pollution. Their ranking on environmental indicators including particulate matter (PM) smaller than 2.5 μm, Ozone, diesel PM and traffic proximity and volume (daily traffic count/distance to road) for both communities are similar. Both communities rank higher than 90th percentile in traffic proximity and volume, i.e., these communities have higher exposure to traffic proximity than where 90% of the US population lives. Both communities also have the same percent of people over 65 and under 5 years in age.

**Table 1:** Percentile ranking for Somerville and Chinatown area compared to all block groups in USA for environmental justice indexes, environmental indicators, and demographic indicators from EPA’s EJSCREEN tool (https://www.epa.gov/ejscreen) [17]

	Somerville	Chinatown
Environmental Justice (EJ) Indexes		
EJ Index for Particulate Matter (PM 2.5)	52	78
EJ Index for Ozone	51	81
EJ Index for NATA* Diesel PM	36	93
EJ Index for NATA* Air Toxics Cancer Risk	50	83
EJ Index for NATA* Respiratory Hazard Index	49	84
EJ Index for Traffic Proximity and Volume	80	99
Environmental Indicators		
Particulate Matter (PM)	13	14
Ozone	26	25
NATA* Diesel PM	80-90th	95-100th
NATA* Air Toxics Cancer Risk	<50th	70-80th
NATA* Respiratory Hazard Index	50-60th	80-90th
Traffic Proximity and Volume (daily traffic count/distance to road)	94	99
Demographic Indicators		
Demographic Index	47	89
Minority Population	51	80
Low Income Population	43	92
Linguistically Isolated Population	80	98
Population with Less Than High School Education	52	93
Population under Age 5	30	31
Population over Age 64	30	31

*The National-Scale Air Toxics Assessment (NATA)

**Fig. 2:**
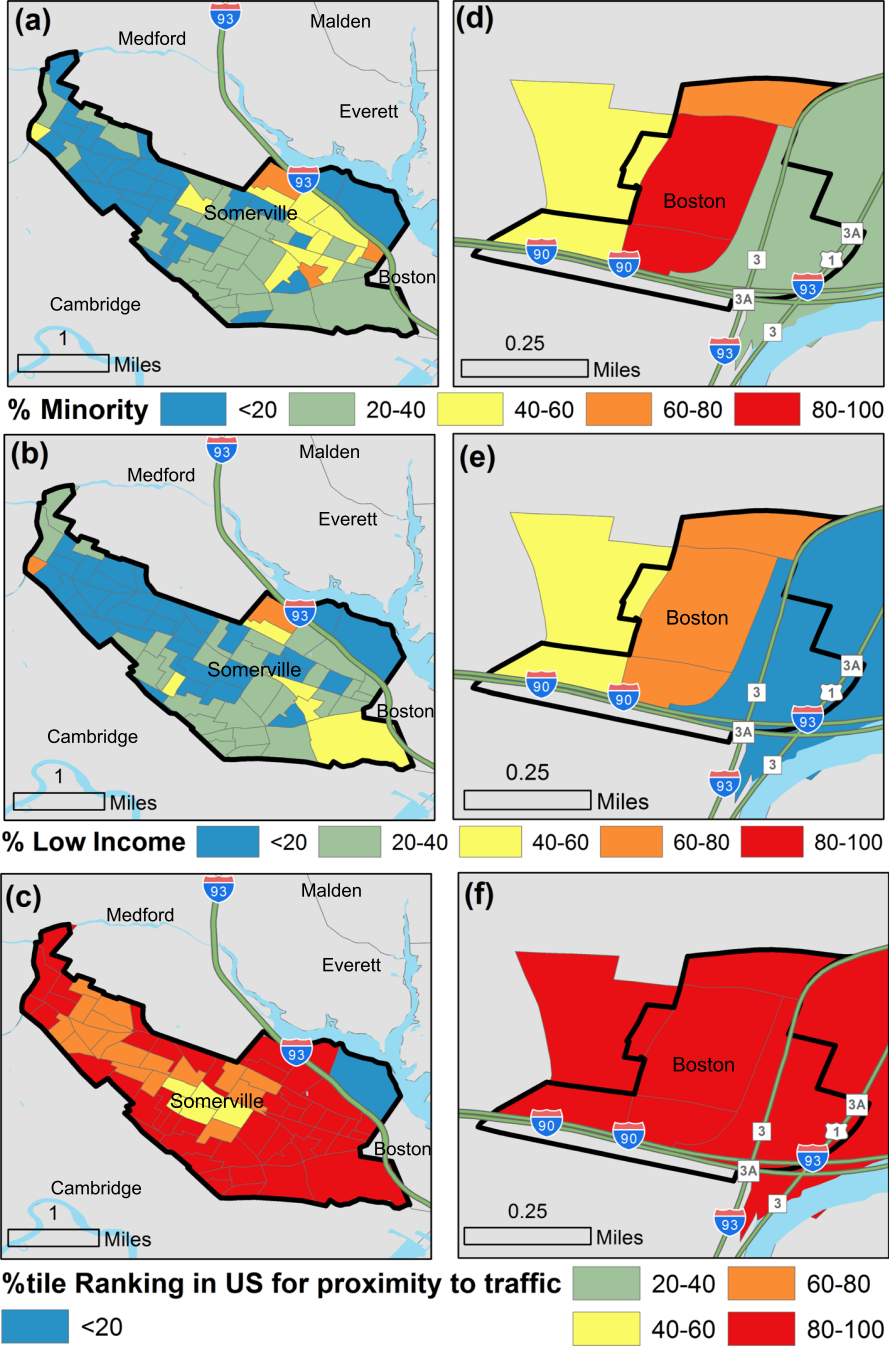
Percentile rankings for block groups in Somerville, MA & Boston, Chinatown compared to block groups in US for two demographic indicators (percent of population considered minority and percent of population considered low-income) [18] and an environmental indicator (proximity to traffic) [19]. data was obtained from US EPA’s EJSCREEN (https://www.epa.gov/ejscreen)

The Chinatown District has a higher percent minority and low-income population overall, however, it should be noted that the segment of Somerville along the highway is home to a higher percent of people of color and low-income households that the city overall as shown in Fig. 2. Figure 2 shows the percentile ranking of the block groups in the two communities compared to all block groups in US for two demographic indicators (percent of population that is considered minority, percent of population considered low-minority) and an environmental indicator (proximity to traffic).

As a first step in this work we set out to explore how each of these two communities were defining and engaging stakeholders in an effort to better understand how each community would translate knowledge into practice. Both communities were included in an effort to understand differences in their approaches to research translation and the ways in which they were or were not successful. As such, this paper specifically explores the research question: what are the approaches to community engagement employed by organizations in Somerville and Chinatown in efforts to mitigate the effects of TRAP? What emerged was a story about the role of political history in present day efforts related to community engagement in public health promotion and policy advocacy, as well as the importance of leveraging existing advocacy efforts and infrastructure. We provide a brief background on the Community Assessment of Freeway Exposure and Health (CAFEH) partnership, a multi-university and multi-community consortium that studies a broad range of TRAP-related issues, and our guiding model, the Interactive Systems Framework (ISF) [20], followed by a detailed description of the research methodology, and a discussion of the study results. Findings contribute to the literature by expanding our understanding of how community-level factors influence the dynamic relationship between science, action and policy practice in communities and municipalities.

### The CAFEH partnership

Between 1956 and 1963 the Massachusetts Department of Transportation (DOT) began construction on the first portion of I-93, which runs 24 miles north from Medford to the New Hampshire border [21]. The section of I-93 that runs south from Medford through Somerville and Boston was completed in 1973 [22]. At the time, both Somerville and Chinatown were both home to large populations of new immigrant, low income and working-class residents. Over the course of planning and construction, state officials and planners were met with opposition from activists and organizers who mobilized large-scale protests in response to community concerns about the adverse effects of the highways on resident health and well-being [23]. Protests were successful in stopping some highway construction, but not I-93, and although there were mitigations promised to Somerville for the highway, they never materialized [22, 23].

In 2006, 33 years after the construction of I-93, local resident organizers who had been actively contesting the highway and examining its health impacts on Somerville residents, initiated a partnership with university researchers, launching a research and action agenda focused on TRAP [24]. CAFEH emerged from this partnership in 2008 and continues today. The resulting body of research focuses on near-highway pollution and health in communities bordered – and some cases divided – by I-93, including Somerville and Chinatown. The community research partnership began with funding from NIEHS with the aim of evaluating the relationship between TRAP exposure and health, increasing the knowledge base to inform policy and the development of mitigation practices. The results of this work were used by the partnership to inform solution-oriented research and community action [24].

Over the course of the CAFEH partnership leadership has shifted between community and academic stakeholders as has the level of engagement. Early on researchers led the effort with guidance from community members, but more recently as the scientific knowledge developed by CAFEH (and other research groups) has matured, community leaders have taken a leadership role in translation of study findings into action through developing protective interventions at both the community and individual level. These shifts have also led to increased disciplinary diversity among the team including a greater role for social scientists, architects and urban planners, among a team that was initially made up primarily of environmental engineering and public health researchers.

Within the original project, US Environmental Protection Agency (EPA) funding provided early critical supplemental support to two of the lead graduate students. Once the partnership was established, the team also developed and submitted proposals for additional research that led to funding from US Housing and Urban Development and the National Heart Lung and Blood Institute [2, 25]. These grants allowed team members to pursue research complementary to their environmental epidemiology work including testing interventions such as air filtration aimed at reducing exposure and health risk [26].

A grant from the Kresge Foundation in 2013 moved the project firmly into addressing policy and practice. This grant was aimed at influencing the City of Somerville to take protective action for housing near the highway and led to considerable engagement with the city and ad hoc review of new construction for ventilation and filtration, but, as yet anyway, not an institutionalized approach [2]. In Chinatown, CAFEH partners influenced planning for a new school campus proposed for a state-owned parcel of land between highway ramps. Influenced by research and community advocacy, Boston Public Schools leaders first incorporated air filtration changes to the proposed school design, then dropped the proposed site entirely, as the result of budgetary concerns. In a similar timeframe to this work, a grant from the National Library of Medicine funded development of an innovative interactive air pollution map that was used in an educational intervention with Chinese immigrants in Chinatown [27]. Most recently funding from NIEHS has supported research and action designed to catalyze the translation of science to practice and, very recently, a randomized crossover trial of portable air filtration in housing near the highway in Somerville. A second Housing and Urban Development funded study is also assessing sustainable air quality practices in affordable multifamily housing near the highway in Somerville, in particular, the filtration efficiency of mechanical ventilation systems.

**Fig. 3:**
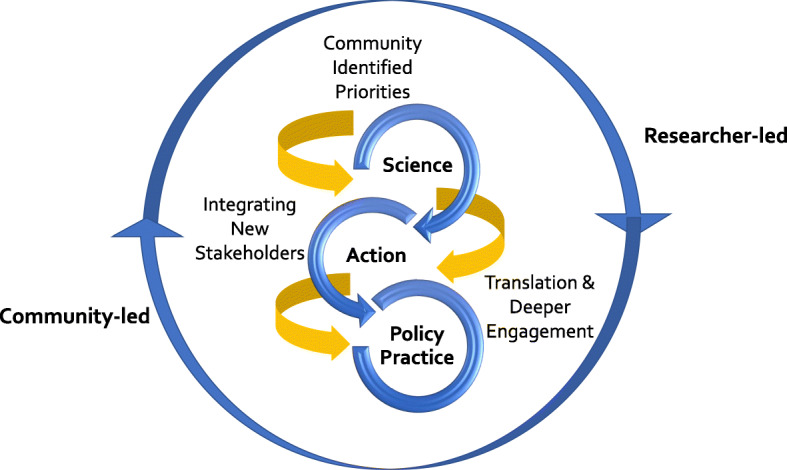
From Research to Action

The trajectory of CAFEH illustrates how community research partnerships shift over time with respect to aims and make-up. As illustrated in Fig. 3: From Research to Action, CAFEH has moved from a focus on scientific research to evidence-based public health action and informing policy-practice. Throughout this process the team has broadened its disciplinary expertise to inform the work, while simultaneously expanding its reach within the community. As a result, team leadership has intentionally rotated back and forth between community and academic investigators dependent on project aims.

The current study, *Near Highway Exposure: Research to Action*, funded by NIEHS, is focused on translating research to local action aimed at mitigating the health effects associated with TRAP exposures, and the study of community-level factors that influence translation. This work is guided by the Interactive Systems Framework (ISF) [20], first introduced by the Centers for Disease Control and Prevention in 2008 to help bridge connections between science and practice with the goal of improving evidenced-based interventions [28, 29]. The ISF includes three components: (1) the Prevention Synthesis and Translation System (2) the Prevention Support System and, (3) the Prevention Delivery System [28]. Together these components are designed to create the conditions for translating new innovation into practice. Community stakeholders are critical to each component of this framework [30, 31].

CAFEH, through community partnership, has generated substantial evidence for association between TRAP and poor health [32–34], specifically, the ultrafine particle component of TRAP. Ultrafine particles are smaller than 0.1 μm in diameter (for comparison, the thickness of human hair is ~ 70 μm), and as a result they can penetrate deep into the body where they can be translocated to various organs. Patton et al. measured spatial and temporal differences in traffic-related air pollution in these neighborhoods and reported ultrafine particle number concentrations of 30,000 (IQR: 49,000) and 26,000 (IQR: 26,000) particles/cm^3^ in near-highway neighborhood in Somerville and Chinatown, respectively [35]. CAFEH research showed that long-term exposures to ultrafine particles were associated with biomarkers of cardiovascular disease risk, specifically in Chinatown and Somerville [32]. This work was among the first to show health associations with exposure to ultrafine particles and was based on painstaking efforts to measure near-roadway gradients of pollution, develop mathematical models to predict exposure, and then assign individualized exposures to residents in the near highway communities [32, 35–37]. Guided by the IFS, we now seek to inform policy and practice in an effort to mitigate TRAP exposure. To inform our work, we first sought to advance our understanding of how community-level factors influence the translation of research into practice to address TRAP exposure.

## Methods

A qualitative instrumental case study design was employed. Instrumental design involves selecting a particular case in order to understand specific issues or challenges [38, 39]. This approach is widely used in social science research and focuses on the study of a case in a real-life, present-day setting [38, 39]. Data include interviews, observations, documents and audiovisual resources [38].

The study draws on three data sources: key informant interviews (*n* = 15), ongoing analysis of weekly meeting minutes and direct observation of research and community meetings (*n* = 10). A summer of data collection methods can be seen in Table 2. All protocols were reviewed by the Boston University Charles River Campus Institutional Review Board, protocol #4434X. Interviews were transcribed verbatim and reviewed by the research team for accuracy.

**Table 2:** Data collection summary

Data Collection Methods	n	Time Point
Key Informant interviews	15	Baseline
Observations	10	Ongoing
Reports	2	Baseline
Meeting summary log	1	Ongoing

### Key informant interviews

Initial interviews with project steering committee members in April to May 2017 and follow-up interviews in December 2018 with key partners and a subset of committee members in Somerville and Chinatown focused on community process. The study was announced at a project meeting and interviews were scheduled by email at a time and location convenient to participants. At the time of the interview a script outlining the procedures (available as a supplementary file), elements of consent were reviewed and participant questions about the study were addressed. A semi-structured interview guide exploring participants’ roles on the project; participation and leadership; perceptions of decision-making, inclusion and engagement; and expectations and goals was used. In addition, community context, outreach and engagement strategies, as well as strategies for community action related to TRAP were examined. Finally, perceptions of community-level factors influencing research translation and potential challenges and levers were queried. Interviews were recorded and transcribed. Transcripts and recordings were then reviewed to verify their accuracy. Participants were contacted for follow-up on an on-going basis over the course of the study. Further, additional interviews were conducted with two Somerville activists who led opposition to the highway in the 1960’s.

### Observations

Observations were conducted from April 2017–December 2019. The overall goal of the observations was to examine dynamics and participation at meetings and public forums associated with the project. Observations were planned in collaboration with the project steering committee. Members of the steering committee introduced the researchers at community meetings and events and explained the goals of the study. Permission was requested to record meetings, when feasible, depending on participants’ comfort with being recorded, the size of the event and the acoustics of the meeting space. At large forums additional note takers were present and introduced to the group. Comprehensive notes were taken during and at the culmination of each observation. Observation notes were typed and then analyzed thematically.

### Document review

Project documents included health lens analysis protocols, charrette processes, weekly meeting plans and agendas, and committee meeting minutes. The researchers engaged in continuous data collection and analysis, and provided feedback to the steering committee periodically.

### Data analysis

Two types of data analysis were employed, holistic analysis of the whole case, and embedded analysis of specific parts of the case [38]. A chronology of events was examined in addition to key themes to assess the complexity of the case [38], two members of the research team independently coded the interview transcripts and developed a codebook. Each data source was coded inductively, focusing on the data itself without preconceived categories [40, 41]. The researchers also reviewed and analyzed community meeting minutes and notes from direct observation of community meetings.

First, the researchers read each interview transcript multiple times to immerse themselves in the data, to search for meaningful patterns [40], and to reflect on their questions and reactions to the codes [42]. The researchers discussed the initial analysis to ensure consistency in their analytic approach [43]. Second, they developed codes using NVivo by labeling and naming selected text. Third, the researchers sorted codes into possible themes and explored relationships between themes and codes across levels [40]. Fourth, the researchers further reviewed and refined the themes to ensure the data within each theme was both cohesive and distinct [40, 41]. Fifth, the researchers identified the larger story within the data in collaboration with participants [40], and elicited feedback from members of the steering committee. In the final phase of analysis, the researchers selected illustrative quotes to produce a succinct, cogent story of the data within and across the identified themes from the three sources of data [40].

## Results

A total of 15 key informant interviews were conducted with project partners and community stakeholders and 10 community meetings focused on TRAP were observed. Interview participants included academic researchers and staff (4), community leaders (5), planners (3), residents (3). Meetings included community planning meetings, informational sessions and planning charrettes. In both communities initial planning meetings were informational and or explored community health related priorities with an emphasis TRAP. Later meetings in Somerville focused on sound walls as a strategy to mitigate TRAP, meanwhile in Chinatown the focus was on buildings and then incorporating TRAP in the Chinatown Master Plan.

Although Somerville and Chinatown fought steadfastly against the construction of I-93, both lost parts of their communities to make room for the highways, and were then left to address the adverse health effects of TRAP. As one participant observed,

*… both of these communities were influenced by the building and development of I-93 and the Mass Pike. Chinatown, half of it was destroyed; Somerville, they fought against the highway and it was built against their wishes. So, in both communities … boundaries … were largely defined by the construction of the highways. It goes way back. It is very deep.*

Yet, despite their shared past, the present-day approaches to public health activism in Somerville and Chinatown are quite different. Our findings highlight the role of contextual factors, namely politics and power, in influencing public health action related to TRAP. Two illustrative case studies are presented. Community context is followed by a discussion of local activism and political power. We then situate each of the communities in context describing their relationship to I-93 as well as strategies for community change and resident engagement past and present, culminating with plans for public health action to mitigate TRAP.

### Somerville

Somerville is a densely populated city located 2 miles northwest of Boston with a population of over 79,000 living in 4.1 mile^2^ [44]. This work has focused on east Somerville which lies adjacent to the highway, and not the western and northern segments of the city. These different areas have different demographics that are quite different. A greater proportion of residents in the near-highway neighborhoods are people of color and foreign-born than the city overall [45]. According to the census, 70% of the population identifies as White, 10% identifies as Hispanic or Latino, 10% Asian, 7% Black or African American, less than 1% American Indian/Alaska Natives and 3% identify as other race [46]. Among Somerville residents, 24.8% (19.8 k people) were born outside the US as of 2017 [44].

Somerville is described as a racially and ethnically diverse community; more specifically, as being home to large numbers of new immigrants. This theme emerged in key informant interviews as well as in meeting minutes as planners talked about the importance of engaging non-English speakers and immigrants. The extensive diversity in Somerville is not apparent at first glance. Outsiders might characterize Somerville as a progressive upper middle-class white community. This runs in stark contrast to the community’s working-class roots, which at the time of economic down turn in the mid-to-late 1900s, earned it the nickname “Slumerville”, when the community was struck by high unemployment rates, disinvestment and political corruption [47].

At the turn of the century Somerville was an epicenter of the new immigrant working class. Ostrander [47] describes Somerville in the late 1800s as a city run by Yankee Aristocrats on the backs of Irish bricklayers. In the early 1920’s the Irish gained political power in Somerville and new immigrants from French Canada, Italy, Portugal and Greece took their place at the bottom of the economic ladder [47]. According to participants, blue collar workers still comprise approximately 33% of the population today:

*… I characterize the population in thirds. I would say one of the thirds is the old blue-collar community, which still lives here. One third is the young educated, but not at this point wealthy, population and then the third is the immigrant population.*

A closer look at the public schools of Somerville today reveals a robust immigrant community with 48.2% of the school children speaking a language other than English [48]. Among the population of 4931 Somerville public school students, 53 languages are spoken [22]. Further, the CAFEH work has been situated primarily in East Somerville neighborhoods, which are closest to I-93 and are substantially more diverse than the city as a whole. In the neighborhoods near I-93, half of the residents are people of color, compared to the city average of 3 in 10 residents of color [22]. Of note, some new immigrant populations, such as Brazilians, are less visible in the census as they don’t fit squarely in US racial and ethnic categories [49]. One participant described the diverse immigrant communities in Somerville:

*… in Somerville [language demographics] are almost equal Portuguese, Spanish, Chinese, Haitian Creole, and there are an awful lot of others here. When people have to move out of town they don’t like it, and many people come back and spend their days or weekends here.*

Rising housing prices associated with redevelopment and gentrification have priced many lower income people out of the Somerville housing market. However, even though they may not be living in the community they return for social activities as well as for church, markets, health services and civic associations [47, 49].

#### Activism and political power in Somerville

In conversations with community partners about the present research and action study, they indicate Somerville has a long history of community activism among residents. Participants noted City residents have worked to protect land use and to limit development expansion in Assembly Square and other neighborhoods:

*… a number of us first started thinking about … participating in how the city evolves, particularly areas with large-scale developments about 20 years ago. Initially, we worked in parallel with city government. We focused a lot at Assembly Square because it is as big as downtown Boston from North to South station and it was largely underutilized after the manufacturing businesses left post WWII. We had citywide meetings, large charrettes, … we were working in parallel with city government … the larger developers … upper hand … After that we got in a series of pretty large lawsuits that we largely wrote ourselves against the four biggest law firms in Boston in real estate and environmental litigation. It took five years, in the meantime we had written zoning and lots of other things. … we ended up winning three fully prosecuted lawsuits in three different court venues on three different principles of law, which took a number of years to play out.*

*… STEP [the Somerville Transportation Equity Partnership, a grassroots coalition] … went to every meeting [Green Line planning meeting] … and put 1500 people in three meetings … the state didn’t want to do the Green Line, they wanted to substitute entirely non-Somerville projects even though the Green Line was the single largest environmental mitigation project for our violations of the Clean Air Act and the state of limitation plan which is how surface transportation relates in federal regulations to the Clean Air Act. So, following that kind of massive unity of citizens [referring to residents at Green Line meetings] and elected officials and others, the state relented and decided to go ahead with the Green Line.*

These are two examples of resident activism in Somerville. The CAFEH partnership itself was the result of years of resident action through the work of the Mystic View Taskforce, which catalyzed Somerville Transportation Equity Partnership. As described by partners, Mystic View Taskforce contested through litigation as Somerville Transportation Equity Partnership actively organized residents, using both capacity building and social action. In their interviews, Somerville partners talk about organizing and working with city officials and state representatives as opposed to working against government.

*When a developer came in and wanted to build a rental building next to my Stop and Shop and we fought to make that condos, and it is going to be condos … it hasn’t been passed yet, it is under consideration. That is one thing we have been working on with the city for a long time, but the city is undergoing a zoning revamp so it is part of that whole process.*

Ostrander (2013) argues residents in Somerville are able to actively engage in political activity through “voluntary associations” (p. 38) because of the absence of a strong elite business class or “urban regime” [47]. This leaves space for the active involvement of residents to influence local decision-making [47]. We also saw this quite clearly in Somerville efforts to engage local leadership in discussions around public health action related to TRAP. Early Somerville meetings included strong participation from local elected officials and state representatives. During these early meetings, political figures spoke about the impact of TRAP and the importance of the research partnership. This participation was largely the result of relationships between community stakeholders and political officials that pre-dated the work of the CAFEH partnership. Leveraging these relationships has benefited the team, as the participation of political leaders has continued across public health action planning meetings in Somerville.

#### Interstate-93, TRAP and public health action

In Somerville, 1950 marked the beginning of neighborhoods being leveled to make way for the “inner belt” of the expressway, this included the “Brickbottom” and States Avenue Neighborhoods [47]. Expansion of I-93 was a public works project that impacted Somerville into the 1970s and residents actively protested. One informant reflected on the political context of resident activism during this time:

*During the period when (a key informant) and her crew were trying to fight I-93, Somerville had incredibly corrupt politics. Everybody was on the take. They had very little influence because of what was going on at the political level. … that was what really screwed things up, decisions were made that didn’t make any sense. There was as much activism in Somerville [about corruption] as there was about fighting the inner belt.*

According to Ostrander (2013), “the city administration is moving away from this private patronage to a ‘good government’ approach” (pg. 38) in an effort to create distance from the corrupt government system of years past [47].

Residents have been fighting I-93 in Somerville for many decades. Another key informant, a lifelong resident of the East Somerville States Avenue neighborhood commonly referred to as “the nunnery” given its proximity to a nearby convent, described the different initiatives residents took on, including marching in the streets with banners, canvassing door-to-door and in the case of her brother-in-law, literally laying down in the mouth of a bulldozer:

*We lost half of our neighborhood on Wisconsin Ave, people moved out because they didn’t want to deal with all the construction going on and didn’t want to wait until their house was gone.*

*We requested I-93 go underground, we were against the elevation. We formed the East Somerville Committee Action group, the first active group in the city; we were civically involved for a long time, and then expanded, this included mostly neighbors, community members. We were protesting, the group stood where the excavation was going to happen. We canvased door to door, except it was too late, the road was planned and construction began.*

This history of highway expansion continues to impact the Somerville community in significant ways. Somerville is the only municipality in Massachusetts that has over 200,000 vehicle miles traveled per day per square mile and the only one with 15,000 diesel trains per year per square mile. A major commuting corridor to Boston, I-93 and two arterials (Routes 28 and 38), together carry 250,000 vehicles per day through Somerville [50]. These pass-through commuters come from outlying municipalities on their way to Boston and Cambridge. While over 25% of Somerville households have no cars and over 50% of residents use public transit or bike or walk to work, residents are subjected to high volumes of traffic and diesel rail pollution because of the seven diesel commuter rail lines traveling through the city, along with heavy highway traffic and the location of the Massachusetts Bay Transportation Authority Commuter Rail Maintenance facility [50].

In 2006 the residents approached researchers to study the health effects of I-93 [51]. The goals of the Somerville partnership with respect to research and action were twofold, to increase awareness and to decrease exposure. One participant shared their vision for this partnership:

*I would like to be able to say that we come up with some real solutions to reduce people’s’ exposure. Technical solutions that are beneficial to people in new housing and old housing. So very concretely those are what I would like to see happen. On a community level in terms of people’s understanding, being able to engage in a way that people learn that one it is an issue they need to be concerned about, but two, that they may have some wherewithal to address these kinds of issues so that it is transferable to other things that people are concerned about. So, building capacity to address needed change, would be something that I would like to see.*

Stakeholders identified both internal and external targets for public health action. Staples (2016) defines targets as individuals or institutions that action groups activate or influence to bring about change [52]. External targets have the decision-making power to help the action group realize its goals, and as internal targets, the community members themselves are engaged in the initiative [51].

Somerville stakeholders are using community development strategies to engage internal targets. These have included information sessions and outreach events. Similar to the community engaged planning process, embedded in the health lens analysis, the charrette process provides residents with data and engages them in discourse, which can build community awareness [53]. Through partnerships with local immigrant serving organizations and English language classes, community partners sought to engage internal stakeholders and build community capacity. These sessions provided background information on TRAP and offered a venue for residents to share their ideas and make recommendations, informing the planning process. One partner shared how the Health Lens Analysis process informs the charrette approach:

*… at the resident level, this is where we are excited about the Health Lens Analysis [it] is a way to have the ultrafine in the conversation, and kind of nesting that within communities’ concerns related to particular projects that may either increase people’s exposure to ultrafines or generate more ultrafine particulates. So, having conversations that blend what community priorities are especially health determinants and how something like ultrafines play into that conversation and using that as a way to have a community lead or at worst community informed response to dealing with the issue.*

These conversations gave participants an opportunity to discuss the most effective targets and identify goals for each target. External targets include municipal leadership and policy makers who have the ability to influence local policy and transportation and highway planning.

*… helping on the formation of policy pieces whether it be guidance or regulation on how understanding of the ultrafine exposure plays into development itself or transportation related decisions or really any land use decisions. Part of it is also bringing it into broader conversations that when people are looking at existing conditions or talking about alternatives or actions, making sure that is a part of a conversation as part of the [Project Partnership Name] piece itself.*

*… while I do think that there is kind of a way to influence up to the state I think most of our focus is kind of our regional scale and going down. Part of it is both kind of municipal leadership, including those who are elected, and municipal staff, like planners, people in community development, people in the engineering or public works side of municipality. So, building awareness but also an actionable awareness around air quality exposure, particularly ultrafine as part of that conversation. So what ways they can alter their work or change what they consider when they look at plans of attack to make change.*

*… the municipal side I think two ways we have come at is are one, especially in Somerville, working with the [Project Partnership Name] group on what a zoning ordinance might look like if it was taking these exposures into account. So that was looking at zoning that might create a district where people might fall into an area that could be impacted by ultrafine particulates and maybe subjecting them to a couple of additional steps to assess whether or not that exposure exists and if so how they would mitigate it. So, doing that within a legislative piece.*

In Somerville, the conversation related to TRAP and public health action has centered on sound walls. Although, sound walls cannot eliminate TRAP, they can reduce exposure [54, 55]. Moreover, those most impacted by TRAP are also impacted by noise. Partners have leveraged their strong ties with local officials as well as the broken promises of the state to propose building sound walls near exposed neighborhoods. Their overall approach to resident engagement draws on a long history of environmental health action. It has been top-down working through partnerships with local officials and state representatives to pressure transportation leaders. They have employed a collaborative community development approach, which began with educating leaders and now residents. They have broadened their campaign to ensure all segments of the community are engaged partnering with local immigrant serving organizations and hosting meetings in Portuguese, Haitian Creole and Spanish.

### Chinatown

Boston’s Chinatown is a historic neighborhood in downtown Boston. I-93 construction through Chinatown was completed in the late 1950s, demolishing homes and cutting the historic Chinese Merchants Association building in half. Massachusetts Turnpike construction then displaced hundreds more residents from 1962 to 1965, just prior to the 1965 liberalization of immigration law, which led to a major increase in the Chinese American population. Today, I-93 and the Massachusetts Turnpike (I-90) together carry 300,000 vehicles per day through Chinatown [51].

Chinatown has experienced successive phases of transformation, first through urban renewal, which razed hundreds of housing units for highway construction, then through expansion of the Tufts University Medical School and Medical Center, and most recently with a massive influx of luxury and market rate high-rise development associated with Boston’s downtown revitalization. Each of these phases has brought increased traffic and development challenges to Boston’s smallest and lowest-income neighborhood.

Chinatown’s city streets are among the most congested in the city, as traffic gridlocks with commuters entering and exiting the highways to work in the downtown financial and commercial district. For decades, the neighborhood’s primary outdoor recreational space has been a basketball court between on and off ramps serving I-93 and I-90. Construction of new housing and a new school immediately next to the highway are ongoing. Unlike some near highway neighborhoods where residents spend large parts of their days away from home, a majority of adults and children (attending the local school which is also right next to I-90) are in Chinatown most of the time on most days. Chinatown was also one of the original CAFEH study areas with high levels of TRAP, including ultrafine particles [51].

*You know, I think the [Project Partnership Name] project over the years it already has increased a lot of people’s awareness of air pollution as an issue. But I don’t know that people really, that other than thinking about it when you open your window, that there is really a lot that people have been able to do about it on an individual level.*

In Chinatown, residents’ concerns about TRAP are situated in the community’s larger concerns about the health of the community, with initiatives focused on practical solutions to benefit individual community members. Chinatown organizing has been primarily focused on community development, specifically affordable housing and resident displacement as a result of gentrification. One key informant reflected on the community’s top priorities:

*I think the biggest challenge is that it [air pollution] is just not the top priority issue for the community. People are concerned about much more immediate issues, like having a stable housing, jobs. But yeah, I think that is probably the biggest difficulty.*

These sentiments were echoed by another key informant who reiterated the interest Chinatown residents share in seeing real changes that benefit the community. Residents are more focused on immediate concerns as opposed to TRAP:

*… there are a lot of more pressing changes that are going on in … Chinatown. I think specifically about the threat of gentrification and displacement, and that a lot of these threats are very imminent, in that people while interested in talking about maybe sound barriers and health disparities, might not be as interested in investing a lot of time to talk about a threat that feels a lot less on their doorstep.*

The community’s focus on the need for real, actionable change including increased access to green space in the neighborhood, was echoed by a key informant:

*I would like to see us make some actual change that has an impact on the Chinese community members that we work with. You know, so maybe making sure that one of the new affordable developments or at least a development with a significant amount of affordable housing has a good air filtration system, or making some changes to the Reggie Wong Park, that would be helpful.*

Elderly residents, in contrast to younger working families, have been the most available and engaged group of Chinatown residents involved in these community initiatives. One informant described the active role elders assume in the community:

*I mean I think that the sector of the community that is probably easiest for us to reach out to and that we have tended to rely on, are the elderly, because the elderly residents, most of them are living in subsidized housing of some type and they are the most active residents in the community because they have time on their hands, they are stable. … whenever you have a communal meeting it is always the elderly that you can really count on them to come out. And you know Chinatown Resident Association it is mixture of generations but it is predominantly those elderly. … we have pretty extensive ties with the home base of active elderly residents, and they are also the most active voters.*

In order to engage residents in CAFEH’s work addressing air pollution, it has been important for community leaders to situate the research in Chinatown’s history. One informant revisited the central role the highway plays in the community’s history and experience of disempowerment:

*I mean I guess we just talk about it in relationship to Chinatown’s history. And you know, because I think the presence of the two highways it is really a part of Chinatown’s history, always being the disempowered community and having land taken from the community and not being protected residents like other neighborhoods. I think we connect it to reinforcing and educating people about the history. And then which is connected, so we try to connect it to other issues, because in a lot of ways we have to understand the history to feel like we have the right to stand up and fight for things….*

Helping Chinatown residents better understand and appreciate the history of the land in their neighborhood has been a central part of community engagement as evidenced by a key informant:

*And if you go back 60–70 years, those were community people’s homes and they were razed during urban renewal. And it was just after urban renewal they gave the land to the highways, and then the land they didn’t give to the highways, there was a small amount of replacement housing built, and the rest of it they gave to Tufts, I think actually most of it was to the Floating Hospital at that time. So that kind of history is important for people to feel like yeah oh we actually do have a right to that land, it is not just that Tufts has a right to this land, we don’t.*

#### Activism and political power in Chinatown

Whereas Somerville is a small densely populated city with its own government structure, Chinatown is one of many distinct neighborhoods in the city of Boston. As such, gaining access to political power has looked quite different. The neighborhood has been historically marginalized, leading community leaders and activists to cultivate a bottom-up, grass roots approach to engaging residents and advocating for the community’s needs [56]. This advocacy has included both community development and social action approaches to community organizing [56]. The community has engaged in a conflictual and contesting relationship with local government officials over time in order to address residents’ needs. More often than not residents have had to contest local power dynamics as a strategy for grassroots change. This long history of social action has contributed to outreach and engagement infrastructure that is highly effective due to strong social capital and community cohesion, as was described by key informants:

*I mean I think the main community assets are it is a fairly organized community, so we already have the resident association, we already have CPA [Chinese Progressive Association, a partner to many [Project Partnership Name] studies]. And there are a few individual tenant associations that we have ties with.*

*I think the tactics that we use are pretty common, like we will use the Chinese media, we will print flyers, every once and a while we will print a newsletter that we might distribute, we do events, like in the summer we do Chinatown block party with a few different organizations and kind of use that as an educational fair. And then you know workshops in the different housing developments as well as community meetings we usually hold in the Quincy school cafeteria. That is where the Residents Association holds its meetings and it is just a really common meeting space. I don’t think there is anything particularly creative about our strategies, I mean sometimes not so much around [Project Partnership Name] but around our Chinatown Stabilization campaign we have done some artistic things too.*

Chinatown partners were highly effective in bringing out diverse intergenerational groups of residents to early community meetings and forums. Residents were actively engaged in discussions about TRAP during early Health Lens Analysis meetings; however, housing and development, as well as safe streets for walkers, preserving cultural resources, and quality open spaces emerged as pressing concerns in meetings in addition to TRAP.

#### Interstate-93, TRAP and public health action

Chinatown, today, remains a minoritized community and although there is a strong political will and high level of resident participation, structural oppression remains. Moreover, historic disinvestment and marginalization coupled with the neighborhood’s central location in the city, have left Chinatown primed for gentrification and displacement. Early conversations about the Health Lens Analysis were focused on thinking about mitigating TRAP in the context of new development. As community conversations progressed the dialogue shifted to a focus on the neighborhood Master Planning process, and, more specifically, how efforts to mitigate TRAP might be built into the Master Plan and the community’s overall strategies for the future. This approach gained considerable endorsement from neighborhood residents as was evidenced by a community planning charrette attended by 90 participants. Also, notable was how truly bilingual (English-Cantonese) it was.

*From being part of other translated meetings, this one felt a more genuine presentation of materials in two languages – with the presentation being generated in Cantonese and facilitators actively trying to make space for non-English speaking residents in small group conversations.*

Through a series of breakout groups, residents participating in the charrette were encouraged to adopt a holistic view of health and visually mapped out how to address community concerns including the need for green space, improved pedestrian safety as well as commercial and residential displacement. Intergenerational collaboration was evident as older residents worked collaboratively alongside younger residents to identify solutions to these concerns. A number of younger residents voiced appreciation for the opportunity to learn more about Chinatown’s history from community elders.

From the perspective of community leaders, the charrette increased awareness of the air pollution issues in relation to health and helped to link air pollution issues with other community concerns related to both health and development. In addition, it was reported that the planning charrette was successful in gaining attention from the city, particularly Boston Transportation Department, The Boston Planning and Development Agency /PLAN: Downtown, and Department of Neighborhood Development. The Boston Planning and Development Agency and Department of Neighborhood Development have sat in on all of the Master Planning Implementation Committee meetings and have been generally supportive of the CAFEH-led Health Lens Analysis and community-led neighborhood plan update – providing data when requested and looking for opportunities to align the community process with theirs.

In sum, both the Somerville and Chinatown cases speak to the importance of political history in shaping approaches to both community engagement overall and public health action. Moreover, the highlight the value of existing community partnership and advocacy infrastructure in shaping the success of community research partnerships. Both communities are organized quite differently in their approaches leveraged past advocacy work to catalyze TRAP-related public health action.

## Discussion

We found the ISF to be a helpful framework for organizing assessing activities related to dissemination activities associated with research translation. In order to support the needs of community partners, *Prevention Synthesis and Translation System*, CAFEH conducted community outreach through English language classes, press releases, community forums, and neighborhood meetings. They also employed educational materials for local dissemination to stakeholders from various community sectors including residents. Despite the benefits of these initiatives, the absence of an internal communications strategy for the dissemination plan was a limitation.

A Health Lens Analysis was then conducted to help the communities examine TRAP through a health lens. Materials developed were used in the context of Health Lens Analysis meetings to engage stakeholders more broadly in TRAP related discussions. This process allowed community stakeholders and residents to think and dialogue about TRAP in the context of community related health and development priorities. At the same time, it was challenging it was challenging because many of the policy and programmatic changes desired will require a long-term effort, and awareness raising conversations occurred without clear pathways for individuals to take immediate protective steps. Nonetheless, the Health Lens Analysis process and associated planning charrettes served as a *Prevention Support System*. Beyond capacity building planning charrettes resulted in digestible community reports and resources available to stakeholders for use in further public health action. Moreover, the team was able to further develop its understanding of interventions and to hone targets and asks for outreach.

We found the notion of the *Prevention Delivery System* did not fully align with our goals as they related to public health action to inform policy practice. This is largely because our partners set out not to implement a specific program but to inform policies at the municipal and state level in an effort to transform practice. Although municipal stakeholders are increasingly sympathetic to and aware of the health impacts of TRAP, there is not a local legislative or regulatory precedent on how to move some of the proposed TRAP-related policies into practice. As such, we found that pairing the ISF with a community organizing framework may serve as a useful approach for examining the dynamic relationship between science, action and policy practice moving forward. This will be critical in ensuring follow up to the Health Lens Analysis process results in shifts in policy practice.

This work is not without limitations. Although multiple forms of data collection were employed including meeting observation, interviews and documents review, the vast majority of baseline interviews were collected with internal stakeholders (i.e., members of the CAFEH research team). Moving forward, interviews with external stakeholders in each community including residents will be critical to understanding the extent to which research findings and the community action that followed have been translated to policy practice. The ISF was helpful in informing the team’s thinking related to systems and structures needed to translate research to practice.

## Conclusions

The current study has important implications for public health action. Public health practitioners need to be aware of the of the diverse sectors that comprise communities as well as interests, priorities and politics both across and with factions of the community. Moving forward, conducting power analyses in the community using a community organizing framework would be beneficial.

## Supplementary Information

**Additional file 1.** Baseline Interview Script was used to collect data from steering committee members and project partners.

## Data Availability

A copy of the interview protocol has been provided. More information about the CAFEH study and available data reports can be found at: https://sites.tufts.edu/cafeh/

## Abbreviations

CAFEH: Community Assessment of Freeway Exposure and Health
CBPR: Community Based Participatory Research
DOT: Department of Transportation
EPA: Environmental Protection Agency
ISF: Interactive Systems Framework
MA: Massachusetts
NIEHS: National Institute of Environmental Health and Science
PM: Particulate Matter
TRAP: Traffic Related Air Pollution
US: United States

## References

[1] Jones MR, Diez-Roux AV, Hajat A, Kershaw KN, O’Neill MS, Guallar E, Post WS, Kaufman JD, Navas-Acien A (2014). Race/ethnicity, residential segregation, and exposure to ambient air pollution: the Multi-Ethnic Study of Atherosclerosis (MESA). Am J Public Health..

[2] Brugge D, Patton AP, Bob A, Reisner E, Lowe L, Bright O-JM, Durant JL, Newman J, Zamore W (2015). Developing community-level policy and practice to reduce traffic-related air pollution exposure. Environ Justice..

[3] Morelli V, Ziegler C, Fawibe O (2017). Environmental justice and underserved communities. Prim Care..

[4] Marshall JD, Swor KR, Nguyen NP (2014). Prioritizing environmental justice and equality: diesel emissions in Southern California. Environ Sci Technol..

[5] Morello-Frosch R, Jesdale BM (2006). Separate and unequal: residential segregation and estimated cancer risks associated with ambient air toxics in US metropolitan areas. Environ Health Perspect..

[6] Sicotte D (2010). Don't waste us: environmental justice through community participation in urban planning. Environ Justice..

[7] Elwood WN, Corrigan JG, Morris KA (2019). NIH-funded CBPR: self-reported community partner and investigator perspectives. J Community Health..

[8] Minkler M, Estrada J, Thayer R, Juachon L, Wakimoto P, Falbe J (2018). Bringing healthy retail to urban “food swamps”: a case study of CBPR-informed policy and neighborhood change in San Francisco. J Urban Health..

[9] Wallerstein N, Duran B (2017). The theoretical, historical and practice roots of CBPR. In: Community based participatory research for health: advancing social and health equity.

[10] Wallerstein NB, Duran B (2006). Using community-based participatory research to address health disparities. Health Promot Pract..

[11] Israel BA, Coombe CM, Cheezum RR, Schulz AJ, McGranaghan RJ, Lichtenstein R, Reyes AG, Clement J, Burris A (2010). Community-based participatory research: a capacity-building approach for policy advocacy aimed at eliminating health disparities. Am J Public Health..

[12] Minkler M (2010). Linking science and policy through community-based participatory research to study and address health disparities. Am J Public Health..

[13] Cacari-Stone L, Wallerstein N, Garcia AP, Minkler M (2014). The promise of community-based participatory research for health equity: a conceptual model for bridging evidence with policy. Am J Public Health..

[14] Birnbaum LS (2009). NIEHS supports partnerships in environmental public health. Prog Community Health Partnersh..

[15] Chino M (2012). Tribal capacity building as a complex adaptive system: new insights, new lessons learned. Int Public Health J..

[16] Nguyen NP, Marshall JD (2018). Impact, efficiency, inequality, and injustice of urban air pollution: variability by emission location. Environ Res Lett..

[17] How to Interpret a Standard Report in EJSCREEN | EJSCREEN: Environmental Justice Screening and Mapping Tool | US EPA https://www.epa.gov/ejscreen/how-interpret-standard-report-ejscreen. Accessed 11 Sept 2020.

[18] Overview of Demographic Indicators in EJSCREEN | EJSCREEN: Environmental Justice Screening and Mapping Tool | US EPA https://www.epa.gov/ejscreen/overview-demographic-indicators-ejscreen. Accessed 11 Sept 2020.

[19] Overview of Environmental Indicators in EJSCREEN | EJSCREEN: Environmental Justice Screening and Mapping Tool | US EPA https://www.epa.gov/ejscreen/overview-environmental-indicators-ejscreen. Accessed 11 Sept 2020).

[20] Wandersman A, Duffy J, Flaspohler P, Noonan R, Lubell K, Stillman L, Blachman M, Dunville R, Saul J (2008). Bridging the gap between prevention research and practice: the interactive systems framework for dissemination and implementation. Am J Community Psychol..

[21] Interstate 93. http://www.interstate-guide.com. Accessed 2019.

[22] Community Assessment of Freeway Exposure and Health (CAFEH) (2019). Noise barriers in Somerville: a health lens analysis.

[23] Crockett K (2018). People before highways: Boston activists, urban planners, and a new movement for City making.

[24] Sprague Martinez L, Reisner E, Campbell M, Brugge D (2017). Participatory democracy, community organizing and the community assessment of freeway exposure and health (CAFEH) partnership. Int J Environ Res Public Health..

[25] Padró-Martínez L, Owusu E, Reisner E, Zamore W, Simon M, Mwamburi M, Brown C, Chung M, Brugge D, Durant J (2015). A randomized cross-over air filtration intervention trial for reducing cardiovascular health risks in residents of public housing near a highway. Int J Environ Res Public Health..

[26] Brugge D, Simon MC, Hudda N, Zellmer M, Corlin L, Cleland S, Lu EY, Rivera S, Byrne M, Chung M (2017). Lessons from in-home air filtration intervention trials to reduce urban ultrafine particle number concentrations. Build Environ..

[27] Wong C, Wu H-C, Cleary EG, Patton AP, Xie A, Grinstein G, Koch-Weser S, Brugge D (2019). Visualizing air pollution: communication of environmental health information in a Chinese immigrant community. J Health Commun..

[28] Flaspohler P, Lesesne CA, Puddy RW, Smith E, Wandersman A (2012). Advances in bridging research and practice: introduction to the second special issue on the interactive system framework for dissemination and implementation. Am J Community Psychol..

[29] Chambers DA (2012). The interactive systems framework for dissemination and implementation: enhancing the opportunity for implementation science. Am J Community Psychol..

[30] Minkler M, Vasquez VB, Chang C, Miller J, Rubin V, Blackwell AG (2008). Promoting healthy public policy through community-based participatory research: ten case studies.

[31] Minkler M, Blackwell AG, Thompson M, Tamir H (2003). Community-based participatory research: implications for public health funding. Am J Public Health..

[32] Lane KJ, Levy JI, Scammell MK, Patton AP, Durant JL, Mwamburi M, Zamore W, Brugge D (2015). Effect of time-activity adjustment on exposure assessment for traffic-related ultrafine particles. J Expo Sci Environ Epidemiol..

[33] Li Y, Lane K, Corlin L, Patton A, Durant J, Thanikachalam M, Woodin M, Wang M, Brugge D (2017). Association of long-term near-highway exposure to ultrafine particles with cardiovascular diseases, diabetes and hypertension. Int J Environ Res Public Health..

[34] Corlin L, Woodin M, Hart JE, Simon MC, Gute DM, Stowell J, Tucker KL, Durant JL, Brugge D (2018). Longitudinal associations of long-term exposure to ultrafine particles with blood pressure and systemic inflammation in puerto rican adults. Environ Health..

[35] Patton AP, Perkins J, Zamore W, Levy JI, Brugge D, Durant JL (2014). Spatial and temporal differences in traffic-related air pollution in three urban neighborhoods near an interstate highway. Atmos Environ..

[36] Padró-Martínez LT, Patton AP, Trull JB, Zamore W, Brugge D, Durant JL (2012). Mobile monitoring of particle number concentration and other traffic-related air pollutants in a near-highway neighborhood over the course of a year. Atmos Environ..

[37] Patton AP, Collins C, Naumova EN, Zamore W, Brugge D, Durant JL (2014). An hourly regression model for ultrafine particles in a near-highway urban area. J Environ Sci Technol..

[38] Creswell JW (2013). Qualitative inquiry and research design: choosing among five approaches.

[39] Crowe S, Cresswell K, Robertson A, Huby G, Avery A, Sheikh A. The case study approach. BMC Med Res Methodol. 2011;11(1):1–9. 10.1186/1471-2288-11-100 PMC3141799 21707982

[40] Braun V, Clarke V (2006). Using thematic analysis in psychology. Qual Res Psychol..

[41] Patton MQ (1990). Qualitative evaluation and research methods.

[42] Charmaz K (2006). Constructing grounded theory.

[43] Boyatzis RE (1998). Transforming qualitative information: thematic analysis and code development.

[44] Somerville, MA. https://datausa.io/profile/geo/somerville-ma/. Accessed 2019.

[45] Assessment of fair housing. https://www.somervillema.gov. Accessed 2019.

[46] 2013–2017 American Community Survey, five year estimates. https://factfinder.census.gov/faces/tableservices/jsf/pages/productview.xhtml?src=CF. Accessed 2019.

[47] Ostrander S (2013). Citizenship and governance in a changing City: Somerville, MA.

[48] School and District Profiles: Somerville. http://profiles.doe.mass.edu/general/general.aspx?topNavID=1&leftNavId=100&orgcode=02740000&orgtypecode=5. Accessed 2019.

[49] Marcelli E, Holmes L, Estella D, da Rocha F, Granberry P, Buxton O (2009). (in) visible (Im)migrants: the health and socioeconomic integration of Brazilians in metropolitan Boston.

[50] Sustainable transportation. https://www.somervillema.gov/sustainaville/sustainable-transportation. Accessed 2019.

[51] Brugge DRE, Durant J, Zamore W, Lowe L, Sprague Martinez LS, Kurtz-Rossi S, Eliasziw M, Keppard B (2016). Near Highway pollution: from research to action.

[52] Staples L (2016). Roots to power: a manual for grassroots organizing: a manual for grassroots organizing.

[53] Lennertz B, Steuteville R, Langdon P (2003). The charrette as an agent for change. New urbanism: comprehensive report & best practices guide.

[54] Baldauf R, Thoma E, Khlystov A, Isakov V, Bowker G, Long T, Snow R (2008). Impacts of noise barriers on near-road air quality. Atmos Environ..

[55] Recommendation for constructing roadside vegetation barriers to improve near-road air quality. https://www.epa.gov/sites/production/files/2016-08/documents/recommendations_for_constructing_roadside_vegetation_barriers_to_improve_near-road_air_quality.pdf. Accessed 2019.

[56] Lowe L, Brugge D. Grassroots organizing in Boston Chinatown. In: Ostrander SA, Portney KE, editors. Acting civically: from urban neighborhoods to higher education. Medford: Tufts University Press; 2007. p. 44–71.

