# Supplementary material for: Two communities, one highway and the fight for clean air: the role of political history in shaping community engagement and environmental health research translation
Source: BMC Public Health. 2020 Nov 11;20:1690. doi: 10.1186/s12889-020-09751-w (PMC7656715; doi:10.1186/s12889-020-09751-w)
Supplement: Supplementary file 1 — Additional file 1. Baseline Interview Script was used to collect data from steering committee members and project partners. [file 12889_2020_9751_MOESM1_ESM.docx]

**Baseline Stakeholder Interviews**

**Introductory**

How did you become connected with the CAFEH partnership?

What are your expectations for the project? (Probe: How are your expectations for the current research an action study different)

**Project Expectations**

What change do you expect to see in the community?

What challenges do you anticipate the group will run into along the way?

What describe the community outreach process you will use?

What tactics and strategies do you anticipate using to achieve the change you would like to see?

What points of leverage (handles) are available to you?  (Probes: individuals, programs, organizations, institutions, policies, resources, values, other)

What community assets and resources will you tap into? (Probes: individuals, programs, organizations, institutions, policies, resources, values, other)

How do you anticipate the community will react?

**Team dynamics and leadership**

How inclusive is the team?

How are decisions made?

To what extent do team members feel a part of decisions?

How would you describe the leadership style?

How are disagreements and conflict handled at the meetings?

How would you characterize communication among the team?

**Meeting Logistics**

What goes well at the meetings?

What needs to be improved at the meetings?

How do team members catch up if they miss a meeting?

Do team members understand what is being said at the meetings?

How comfortable are team members asking questions at the meetings?

How comfortable are team members speaking openly and honestly at the meetings?

To what extent are team members listened to at meetings?

To what extent are team members respected?
